# Simulated Clinical Encounters Using Patient-Operated mHealth: Experimental Study to Investigate Patient-Provider Communication

**DOI:** 10.2196/11131

**Published:** 2018-11-01

**Authors:** Harry Tunnell, Anthony Faiola, Davide Bolchini, Rebecca Bartlett Ellis

**Affiliations:** 1 School of Informatics and Computing Department of Human-Centered Computing Indiana University Indianapolis, IN United States; 2 Department of Biomedical and Health Information Sciences University of Illinois at Chicago Chicago, IL United States; 3 School of Nursing Indiana University Indianapolis, IN United States

**Keywords:** medical informatics, personal health record, medication reconciliation

## Abstract

**Background:**

This study investigates patient-centered mobile health (mHealth) technology in terms of the secondary user experience (UX). Specifically, it examines how personal mobile technology, under patient control, can be used to improve patient-provider communication about the patient’s health care during their first visit to a provider. Common ground, a theory about language use, is used as the theoretical basis to examine interactions. A novel concept of this study is that it is one of the first empirical studies to explore the relative meaningfulness of a secondary UX for specific health care tasks.

**Objective:**

The objective of this study was to investigate the extent that patient-operated mHealth technology can be designed to improve the communication between the patient and provider during an initial face-to-face encounter.

**Methods:**

The experimental study was conducted in 2 large Midwestern cities from February 2016 to May 2016. A custom-designed smartphone app prototype was used as the study treatment. The experimental design was posttest-only control group and included video-recorded simulated face-to-face clinical encounters in which an actor role-played a patient. Experienced clinicians consisting of doctors (n=4) and nurses (n=8) were the study participants. A thematic analysis of qualitative data was performed. Quantitative data collected from time on task measurements were analyzed using descriptive statistics.

**Results:**

Three themes that represent how grounding manifested during the encounter, what it meant for communication during the encounter, and how it influenced the provider’s perception of the patient emerged from the qualitative analysis. The descriptive statistics were important for inferring evidence of efficiency and effectiveness of communication for providers. Overall, encounter and task times averaged slightly faster in almost every instance for the treatment group than that in the control group. Common ground clearly was better in the treatment group, indicating that the idea of designing for the secondary UX to improve provider outcomes has merit.

**Conclusions:**

Combining the notions of common ground, human-computer interaction design, and smartphone technology resulted in a prototype that improved the efficiency and effectiveness of face-to-face collaboration for secondary users. The experimental study is one of the first studies to demonstrate that an investment in the secondary UX for high payoff tasks has value but that not all secondary UXs are meaningful for design. This observation is useful for prioritizing how resources should be applied when considering the secondary UX.

## Introduction

### Statement of the Problem

The ubiquity of smartphones gives patients access to many apps that can potentially support health care needs. These types of technologies allow patients to track and trend various health behaviors that they may subsequently want to share with health care providers. In this context, health care providers become what is known as *secondary users*, a term from the human-computer interaction (HCI) discipline that reflects a type of user who is affected by the main or primary user’s operation (ie, the patient) of a technology. [1-3]. Primary users, on the other hand, are the dominant operators that control the system and the dissemination of its information and are patients in this context [1]. Some researchers believe that the secondary user experience (UX) has the potential to improve collaboration and satisfaction in a variety of settings and, as such, advocate for its inclusion in interactive systems design [4-6]. However, little is known about the experience of the secondary user, especially in the context of health care provider encounters.

Recently, it has become common for more people to access the internet through mobile devices than personal computers [7]. The advent of always-internet-connected mobile technologies portends a wider set of UXs than previously envisioned. The lack of any practical geographic or temporal restrictions on the use of some types of patient-centered health information technology (HIT), such as a smartphone personal health record (PHR) app, is a recent phenomenon that impacts users and their experiences. Anytime someone acts with an interactive system through an interface in public, he potentially creates a UX for others; this is the secondary UX. With more than 165 million smartphone users in the United States as of 2014, secondary UXs have the potential to become routine [8].

The ubiquity of smartphones gives patients access to technologies that can support their health care needs in ways previously unavailable to lay people. Pew Research reported that within a 1-year period, 62% of US smartphone owners used their devices to look up health information [9]. The ability of smartphones to store and manage information allows patients to track and trend health data and share these data with health care providers in a manner that informs clinical decision making. It is in this context that we believe health care providers are interesting as secondary users of patient-controlled devices. In light of this assessment, we decided to investigate patient-centered mobile health (mHealth) technology in terms of the secondary UX. Specifically, this study examines how personal mobile technology, under patient control, can be used to improve patient-provider communication about the patient’s health care during their first visit to a provider.

A novel concept of this study is that it is one of the first empirical studies to explore the relative meaningfulness of a secondary UX for a specific task. Not all UXs are likely to have value during task-oriented communication. In health care, patient health often relies on successful collaborations between patients and providers [10]. Because there are often common tasks for specific types of encounter, for example, a first face-to-face encounter between a provider and new patient, such tasks can be anticipated and designed for during a user-centered technology design process. However, before designing technology interventions for such tasks, it is important to understand which tasks benefit most from a technology solution. This study, through its emphasis on meaningfulness, provides a model for evaluating secondary UX value for a communication.

Secondary users have been identified in the medical informatics literature but operationalized in terms of patients. This study introduces the opposite phenomenon and looks at secondary users as providers. It is in this context that we explore how a patient as a primary user can manage technology in a way that improves outcomes for providers who are secondary users.

To meaningfully improve HCI design for secondary users, we must take into consideration the dynamic interplay between both types of users. This study does this by investigating secondary UXs according to common ground, a communication theory about language use. Overall, 2 assumptions underpinning this research are that (1) the creation of common ground is a key element of the secondary UX that contributes to improved communication and (2) interfaces for interactive systems can be designed to facilitate the creation of this key element.

The user type most often researched is the primary user, and investigations about secondary users are limited [11]. As the HCI literature about secondary users is sparse, technology is most often understood from the perspective of one user type. In fact, Inbar and Tractinsky [12] reported that secondary users are missing from both the theoretical and practical perspectives in HCI. Consequently, there is little research that exists to empirically demonstrate why secondary users should be included as a consideration in the design of technology. The currently available research merely indicates that secondary UXs exist and that secondary users are a relevant stakeholder group [3,11,13]. Health care, and the increasing emphasis on patient-centered technology, is an especially vital context in which to study the secondary UX because PHR apps are an example of HIT where one person’s use creates UXs for others.

Another shortcoming in the HCI literature about secondary users is the lack of a theoretical basis to explain why secondary users are relevant and should be considered in technology design. We believe that common ground can fill this void. Common ground, a component of effective collaboration, is established when people have certain knowledge in common and know that they have this knowledge in common [14]. Our study revealed a gap in health care knowledge between patients and providers that can potentially be bridged with the aid of technology [15-17]. Such a knowledge disparity is not surprising, as patients are lay people and providers are experts regarding health care, which can make the attainment of common ground between them elusive during an encounter. The short duration of encounters can further constrain communication and information sharing, also making common ground difficult to obtain.

Although improving patient access to health information leads to increased participation of patients in health-related decision making, it is not merely the access to data that creates this impact [18]. The authors believe that it is an improvement in *common ground* between patients and their providers that makes superior patient engagement possible. Technology designs that reduce ambiguity in communication between patients and providers have the potential to improve common ground.

### Theoretical Model

Common ground theory provides the theoretical model for this study. Common ground is defined as “a proposition *p* is common ground if: all the people conversing know *p* and they all know they know *p* ” [19]. The theory was developed by Clark [20] and explains how people achieve sufficient shared knowledge to successfully complete a communication. *Grounding* is the process to make communication effective and common ground is created because of the grounding process [21].

During face-to-face meetings, the ability to share content with someone can be limited by the lack of interactivity of typical physical tools (eg, pen and paper) used by team members [22]. One of the attractions of common ground for secondary UX research is the idea of external representation. External representation is a way to represent components of the communication in physical form. Clark [20] provided the example of a chess game with the board and pieces serving as external representations of the players. The position of the players on the board unambiguously shows the current state of the game.

In HCI, a smartphone interface that displays objects of interest to primary and secondary users is an external representation. Using the chess example once more, the board and chess pieces are digitally represented in the interface, again unambiguously showing the current state of the game. Direct manipulation interfaces (eg, graphical user interfaces) have provided excellent support for grounding for many years because of their capacity to continuously represent objects of interest and provide feedback about the effect of actions [23]. Thus, the relation between computer interfaces and grounding is already established and does not require additional explication for this study.

### Constraints on Grounding

Actions are important components of common ground. A joint action occurs when people intend to do their parts in the communication and believe that the joint action includes their parts and the parts of the other participants [19]. Common ground is incrementally built based on the history of joint actions [24]. When joint actions are mediated by interactive systems, the technology places constraints on the establishment of common ground. Constraints are often considered as a negative attribute. However, in this context, constraints are *positive* for grounding because they reduce ambiguity [19]. The more constraints supported by a technology because of different combinations of devices and interfaces, the better [24]. There are 8 constraints for grounding (Table 1).

Constraints can be used to predict the problems that people will have with an information system by evaluating which constraints are present or absent when using the system [19]. The concept of constraints means that it is possible to anticipate what the UX will be with a product for a particular user type. As the secondary UX can be anticipated through the evaluation of the constraints, it can be designed for during product development. The ability to predict the experience means that common ground theory can be used to explain problems that people have with an information system in certain contexts [25].

### Constraints and Smartphones

Unlike most communication technologies, smartphones can support all constraints in a face-to-face setting. Table 2 shows comparisons of communication mediums and constraints (X is a supported constraint). The fact that smartphones allow users to switch back and forth between functionalities (eg, email or short message service [SMS] text messaging) seamlessly is an exciting prospect and important for grounding. For example, smartphone users do not have to find and go to a fixed system or workstation to send and receive nontelephonic messages because they have smartphone apps that perform several communication functions.

**Table 1:** Constraints on grounding.

Constraint^a^	Description
Copresence	When A and B are colocated, such as in the same room
Visibility	When A and B can see each other
Audibility	When A and B talk to each other
Contemporality	When B receives messages at about the same time that A produces them
Simultaneity	When A and B can send and receive messages simultaneously
Sequentiality	When A’s turn and B’s turn cannot occur out of sequence
Reviewability	When B can review A’s messages, as in written communication
Revisability	When A can revise messages for B before they are seen by others (outside of A and B)

^a^Adapted from Grounding in Communication by Clark and Brennan [21].

**Table 2:** Constraints on communication comparison chart. X refers to a supported constraint.

Medium	Copresence	Visibility	Audibility	Contemporality	Simultaneity	Sequentiality	Reviewability	Revisability
Face-to-face	X	X	X	X	X	X		
Telephone			X	X	X	X		
Video tele-conference		X	X	X	X	X		
Letters							X	X
Email or text							X	X
Mobile phone^a^	X	X	X	X	X	X	X	X

^a^Mobile phone as a medium added to table. Table adapted from Grounding in Communication by Clark and Brennan [21].

**Table 3:** Collaboration mechanics.

Category^a^	Description (mechanics)
Explicit communication	Planned and intentional communication (speaking, writing, gesturing, combining verbal and gestural, and manifesting actions)
Information gathering	Gathering information in shared workspaces from others and their activities (basic group awareness, feedthrough, consequential communication, visual evidence, and overhearing explicit communications)
Management of shared access	Managing group access to objects within the workspace (obtaining a resource, reserving a resource, and protecting work)
Transfer	The movement of objects and tools between people (handoff and deposit)

^a^Adapted from Task Analysis for Groupware Usability Evaluation: Modeling Shared-Workspace Tasks with the Mechanics of Collaboration, by Pinelle et al [27].

The potential to improve how language is used during the patient-provider encounter and thus increase the efficiency of communication between members of the dyad, makes the notion of common ground salient when examining primary and secondary user collaborations. Research suggests that well-designed collaborative technologies speed up the development of common ground by allowing teams to share knowledge, manage actions, and make decisions efficiently [26]. Fortunately, a model to define working with technology in the context of common ground exists. Collaboration mechanics (Table 3), the development of which was influenced by Clark [20] and his work on common ground theory [27], can be used to model collaboration with technology. Therefore, the relations among collaboration mechanics, common ground, and secondary user satisfaction are examined in the experimental study.

### Research Questions

**Figure 1:**
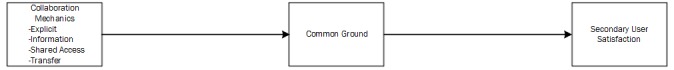
The conceptual model for the research questions.

Although the HCI literature acknowledges that secondary UXs can occur, it does a poor job of revealing how to evaluate the importance of them for an interaction. It is the authors’ contention that the value of secondary UXs in a unique context should be identified to assess its merit for design. Once this is understood, it becomes possible to make an informed decision about how a secondary UX may or may not improve communication. Common ground provides the vehicle in this research to evaluate the quality and importance of a secondary UX during a clinical encounter. The research questions emphasize how common ground is used to demonstrate the relation of language to digital systems, in a manner that results in better communication in an envisioned health care setting. The conceptual model for the research questions is shown in Figure 1. The research questions for this study are as follows:

1. To what extent do smartphone apps designed using collaboration mechanics support grounding (the process of creating common ground) between primary and secondary users during face-to-face collaborations?
2. To what extent do smartphone apps designed to support grounding impact the satisfaction of secondary users during face-to-face collaborations?

## Methods

### Design

The experimental design was a two-group randomized experimental study with posttest measures. The study explored the effect of a prototype HIT compared with a control group (no HIT) on common ground and secondary user satisfaction in a simulated clinical encounter using both qualitative and quantitative methods. A custom-designed HIT was prototyped for use as the experimental treatment. Patient-provider communication was investigated during the performance of the following 3 tasks: problem identification, discussing a patient’s medical history, and medication reconciliation.

### Setting and Sample

**Figure 2:**
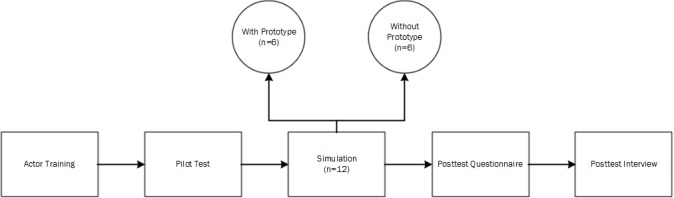
Model of the experimental design.

The study was conducted between February and May 2016 in 2 large Midwestern cities. This study used simulated face-to-face clinical encounters using a trained patient actor. Experienced clinicians consisting of doctors and nurses were the study participants. Participants were recruited using snowball sampling and email solicitations. All participants received monetary compensation. An adult male, with physical characteristics closely matching the scenario and more than a decade of professional and community theater acting experience, was hired (with monetary compensation) to role-play the patient. The same actor was used for all simulations. See Figure 2 for a model of the experimental design.

### Participants

#### Materials and Procedure

The custom-designed HIT prototype used as the treatment for the study was a smartphone PHR app. A PHR is a private, secure, electronic, and Web-based tool that people can use to communicate with their providers and access, manage, and share their personal health information [28-30]. PHRs are examples of HIT with primary (ie, the patient) and secondary (ie, the provider) users, the 2 stakeholders with different knowledge and priorities who need to collaborate to make treatment decisions. Extensive preliminary work before the study was performed to create the prototype as an mHealth platform to support both types of users [15,17].

#### Development of the Prototype

The prototype was developed as a WordPress website so that it could be accessed regardless of smartphone manufacturer and operating system. The personal smartphone of the trained patient actor, a Samsung Galaxy Prevail LTE Android Version 4.4.4, was used to access the prototype during all simulations. The trained patient actor used his own personal smartphone with the prototype installed to minimize training requirements for using the custom-designed HIT prototype during the simulation.

**Figure 3:**
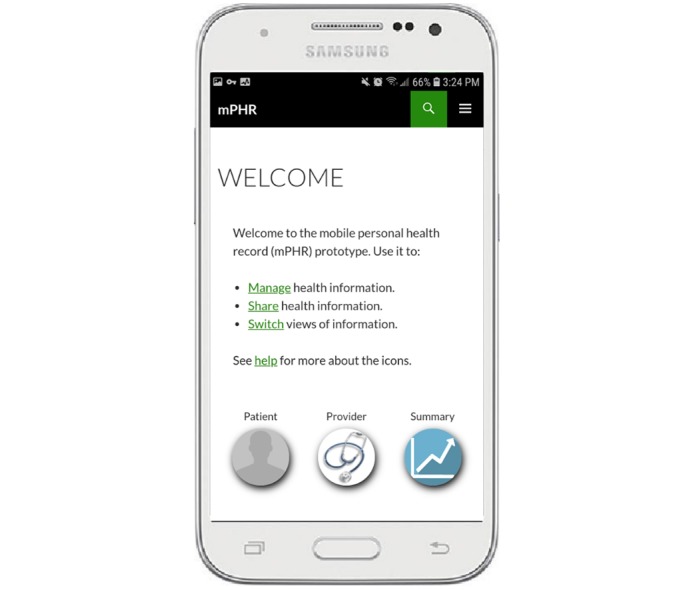
Example Samsung Galaxy Prevail LTE showing the patient and provider buttons. The screen background provides an additional visual cue to users about where they are in the interface. Primary user screens have a white background and secondary user screens a gray background.

The prototype incorporated buttons for patients and providers at the bottom of each page (Figure 3) as a switching mechanism so that tailored views of information contained within the prototype were available for primary and secondary users. Before sharing information with providers, the trained patient actor selected the provider button, which changed the view from his patient view to a context tailored for providers. These different views of information were necessary to improve communication and enable grounding [15].

#### Simulation Scenarios

Both the control and experimental groups participated in a simulated clinical encounter. The scenario required that a provider see a patient for the first time (Multimedia Appendix 1). The reason for the patient’s visit was that he had suffered a rash, which had healed and was no longer visible. As part of the scenario, the provider was required to perform the following 3 tasks with the trained patient actor: problem identification, take a medical history, and medication reconciliation. Overall, 4 medications (Table 4) were part of the patient profile and were expected to be reviewed during medication reconciliation.

**Table 4:** Scenario patient medication profile.

Medication	Dose	Frequency	Reason
Metformin ER	500 mg	Twice/day	Diabetes
Lisinopril	10 mg	Once/day	Blood pressure
Atorvastatin	80 mg	Once/day (night)	Cholesterol
Glipizide	2.5 mg	Three/day with meals	Diabetes

#### Development of Simulation Scenarios

The orientation and training of the trained patient actor was extensive. The trained patient actor reviewed Web-based videos of simulated clinical encounters that included the tasks required for role-play. After reviewing the videos, a rehearsal training session with an experienced (13 years) registered nurse (RN), who has a PhD (Nursing Science) and is an assistant professor of nursing at a large Midwestern university, was conducted. After the session, the simulation documentation was revised and a checklist to assess treatment fidelity was created (Multimedia Appendix 2). The checklist enhanced the internal and external validity by ensuring that the trained patient actor addressed standardized areas with providers and ensured that the study could be replicated [31]. A second rehearsal using the treatment fidelity checklist as a guide was telephonically conducted. The simulation scenario was then pilot-tested for feasibility of study procedures. For this feasibility demonstration, we recruited 1 doctor in the control group and 1 advanced practice registered nurse (APRN) in the treatment group. Finally, a review of the pilot test simulation videos with the trained patient actor was completed before beginning the study.

#### Control Group Simulation

**Figure 4:**
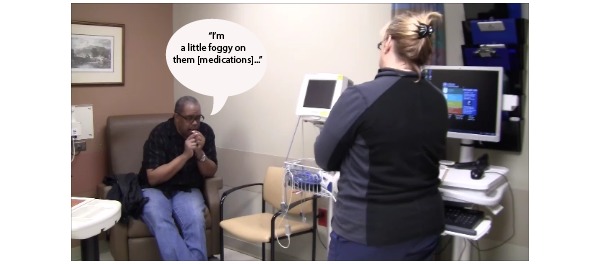
The patient attempts to recall from memory the details of specific medications during the medication reconciliation task.

For the control group, the trained patient actor simulated real patient behavior during a typical encounter by relying on memory to share information with the study participant about the problem that sparked the visit (rash), medical history, and current medications. The medical history for the scenario included multiple ailments: type 2 diabetes mellitus, obesity, hypertension, and high cholesterol. See Figure 4 for an example of the interaction between the trained patient actor and study participant during a control group simulation.

#### Treatment Group Simulation

**Figure 5:**
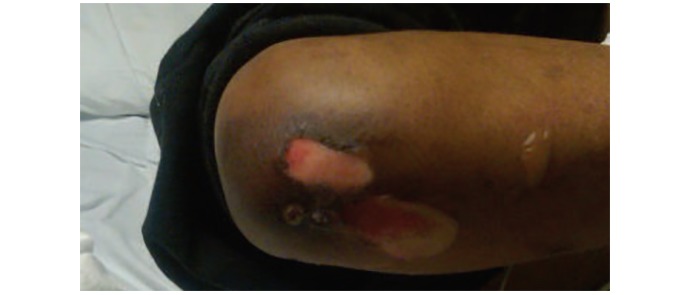
Image used in the prototype to show the patient’s past ailment. Note. Image adapted from Bullosis diabeticorum: Rare presentation in a common disease, by Gupta V, Gulati N, Bahl J, Bajwa J, and Dhawan N, 2014, Case Reports in Endocrinology, p. 2.

The treatment group was shown an image of the rash (Figure 5) by the trained patient actor. A verbal description of the ailment was also provided by the trained patient actor. The image used for the rash was of a patient with *bullosis diabeticorum*, a blistering condition that heals in a few weeks.

**Figure 6:**
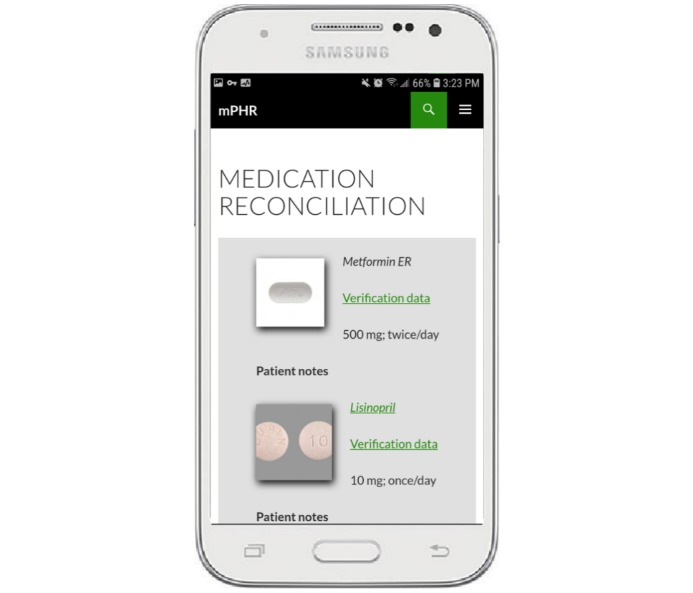
Example Samsung Galaxy Prevail LTE showing the provider view of the medication screen. The screen background provides an additional visual cue to the user about where they are in the interface. Primary user screens have a white background and secondary user screens a gray background.

For medication reconciliation, details about the medications were shared with the treatment group from the prototype (Figure 6) by the trained patient actor. For the medical history tasks, the prototype was not offered to members of the treatment group unless they specifically made a request to look at the information on the device (there were no provider requests to review medical history on the prototype). Before sharing information from the prototype with participants, the trained patient actor switched the view of information from the primary user’s view to the secondary user’s view.

#### Variables

The independent variable was the presence or absence of the prototype. The dependent variables were common ground and secondary user satisfaction. The participant’s perception of performance was also measured because of its likely impact on satisfaction. Common ground and the remaining variables were measured by analyzing the simulation videos, Likert scale responses, and interview transcripts. The details of the 3 measures to evaluate the presence or absence of the treatment are as follows:

1. Recordings: The simulation was video recorded for later analysis with NVivo 11 (a qualitative data analysis software package).
2. Questionnaire: Upon the conclusion of the scenario, the participants completed a 7-point Likert scale questionnaire, which was composed of demographic questions and a set of 15 psychometric scales measuring satisfaction, common ground, and performance (Multimedia Appendix 3).
3. Interviews: Semistructured interviews were audio-recorded and conducted with each participant after the simulation (Multimedia Appendix 4). The results were transcribed using the *TranscribeMe!* service provided through NVivo 11. The transcriptions were *clean verbatim* in which filler words (eg, umm, ah, and you know) were removed. Irrelevant concluding remarks were deleted (eg, closing courtesies). The transcriptions were of good quality. When errors were discovered, they were reviewed, cross-checked, and corrected based upon the audio recordings.

During the study, an emphasis was placed on collecting rich qualitative data, which is gained when saturation is reached. The study sample size was considered adequate because saturation had been reached during earlier phases of this project with similar-sized groups. In addition, saturation was, in fact, achieved during the experimental study. A statistical power analysis indicated that the sample size was too small for hypothesis testing [32]. In lieu of this type of testing, descriptive statistics were used to compare interactions with and without the presence of the prototype. These factors, when taken together, ensured adequate investigation of the dependent variables.

### Procedure

The study was performed at a location selected by the study participants. Hospital treatment rooms and administrative or public spaces in medical facilities and academic buildings were typical. At the beginning of each iteration, the participant was asked to review the study information sheet. The scenario was reviewed with the participant, who was then provided with a pen, clipboard, and paper for taking notes during the simulation. Subsequently, the simulation began, with the use of video recording.

Due to the different specialties and practices encountered during the study, it was impossible to anticipate the flow of the conversation during each simulation. The trained patient actor was authorized to incorporate real-life experiences as necessary to maintain realism during the simulation. For example, discussions about diet and exercise mimicked the trained patient actor’s real-life experiences.

After each simulation, the trained patient actor’s performance was reviewed and behaviors corrected as necessary. The potential effect of actor learning was controlled through training, the use of checklists for treatment fidelity, and counterbalancing between the control and treatment groups [31,33]. Consistency was maintained as much as possible. Minor errors completed by the actor did not disrupt the study as long as the actor was consistent throughout the duration of the simulation.

### Qualitative Analysis

A thematic analysis of the videos and semistructured interview transcripts was performed using NVivo 11. This form of analysis was preferred because it is the most common approach for evaluating qualitative data in health care research [34]. The videos were coded by importing them into NVivo 11, reviewing each video within the software, selecting parts along the range of the media using the software tools, and assigning the range to an appropriate coding container. Summary transcriptions of the videos were also created within NVivo 11, showing general topics of conversation at selected points in the video (eg, medication reconciliation or problem identification). The audio-recorded, semistructured interviews were transcribed, as described earlier, and coded within NVivo 11.

There were 3 rounds of coding, with the first round being conducted manually within the software. During this round of coding, concepts were merged, revised, and discarded as patterns became more obvious. The second round relied on the analytical software tools of NVivo 11 to analyze results from the first round of coding and create a basic concept map. The map was used to sort the concepts that resulted from the first round of coding and aid in reorganizing them into a hierarchical system for analysis. The third round of coding scrutinized the data based on membership in the control or treatment group and evaluated how the content diverged thematically between the groups. A matrix query by assignment to experimental group was run to display the frequency of the coding in each major category of joint action. This round of coding was used to break out details of the data to satisfy the research questions. As a result, themes for the control and treatment groups were identified. The analysis revealed that both groups accomplished a substantial level of grounding for the medical history task. Consequently, it was not coded in detail because the analysis of the task, in and of itself, would not contribute to answering the research questions.

### Quantitative Analysis

Descriptive statistics were employed because of their importance for inferring evidence of communication efficiency for the providers. Descriptive statistics of means, SDs, maximums, and minimums were calculated using SPSS 23. The posttest questionnaires were also evaluated using SPSS 23.

Each video was studied to assess the amount of time spent on the 3 scenario tasks (ie, problem identification, medical history, and medication reconciliation) and the percentage of time the tasks consumed for the entire encounter. The efficiency of the encounter was assessed based on the descriptive statistics for time spent on the 3 required tasks and the overall duration of encounters. The main effectiveness measure of encounters between the control and treatment groups also relied on descriptive statistics. In this case, they were used to evaluate the amount of time spent on patient education, which providers throughout the research identified as an important task to perform with patients. Finally, descriptive statistics were used to analyze the posttest questionnaires and compare responses between the control and treatment groups.

## Results

### Introduction

Overall, 13 nurses and doctors participated in the experimental study, with 12 included in the final analysis. One participant became frustrated during the simulation and requested that the video recording cease. The subsequent interview revealed that she normally used an electronic medical record (EMR), which is an electronic version of a patient chart, and followed a standardized routine when interviewing patients (an EMR was not provided as part of the scenario). The lack of an electronic tool made her feel as if she was not providing quality service to the trained patient actor, which caused her to become frustrated and culminated in her request to cease the video recording. Consequently, these results were excluded from the final analysis.

The backgrounds of the final 12 participants, by assignment to control group and treatment group, are shown in Table 5. Of these, 2 participants were educators and no longer interacted with patients; 1 had transitioned to teaching within the 6 months before the study and the other had 19 years of prior nursing experience. A third participant had shifted to case management within 6 months before the study and had more than 25 years of experience in nursing. The 3 participants had the necessary skills to perform the scenario tasks and were qualified for the study.

The professional experience of the participants varied from less than 1 year to more than 25 years (Table 6). All participants were familiar with the basic technologies used in the study. Each participant owned a smartphone and had 11 or more years of experience using computers. Overall, 9 of the 12 participants reported owning tablet computers.

**Table 5:** Background of participants (12 participants).

Group		Practice or specialty
**Control group**	
	APRN^a^	Geriatric
	APRN	Geriatric
	Doctor	Internal medicine
	Certified nurse midwife	Midwifery
	Registered nurse	Intensive care or intravenous team
	Doctor	Internal medicine
**Treatment group**		
	Doctor	Pulmonary medicine
	Registered nurse	Case management
	Registered nurse	Clinical education
	APRN	Internal medicine
	Registered nurse	Perioperative nursing
	Doctor	General surgery

^a^APRN: advanced practice registered nurse.

**Table 6:** Demographics of participants (12 participants).

Variable and attribute		Control group, n (%)	Treatment group, n (%)
**Gender**			
	Male	2 (17)	1 (8)
	Female	4 (33)	5 (42)
**Age in years**			
	25-34	1 (8)	0 (0)
	35-44	3 (25)	3 (25)
	45-54	2 (17)	3 (25)
**Years of experience**			
	<1	1 (17)	0 (0)
	1-5	3 (25)	0 (0)
	6-10	2 (17)	1 (8)
	11-15	0 (0)	0 (0)
	16-20	0 (0)	1 (8)
	21-25	0 (0)	2 (17)
	> 25	0 (0)	2 (17)

### Themes

Overall, 3 themes emerged from the thematic analysis. The themes ultimately represent how grounding manifested during the encounter, what it meant for communication during the encounter, and how it influenced the provider’s perception of the patient. The themes are discussed below.

- *The patient is engaged in his own health care*. If a patient is willing to take the time to manage his health information electronically, then it is an indication that he is engaged in his own health care.
- *The information is trustworthy*. The use of sophisticated technology by patients implies a higher trustworthiness of information—or, at least, the same level of trustworthiness as other traditionally accepted (but low-use) methods (eg, bringing medication bottles to an appointment).
- *There is enough information at an acceptable level of quality for some level of decision making to be obtained*. This amount of information, and its quality, does not mean that there is a diagnosis. It means that better planning is performed with the patient, even if that plan does nothing, because everything appears to be on track.

In addition to the basic scenario tasks, patient education emerged as a desired discussion topic with patients in the posttest interviews for most participants. As patient education emerged as a topic of broad concern to participants, patient education discussions about the rash, medical history, and medications were coded for the time analysis (see Quantitative Analysis section for these results). Time spent on general nutrition, health and fitness, and similar counseling was not evaluated.

### Efficiency

In general, the trained patient actor’s use of the smartphone seemed to improve provider’s patient care efficiency during the encounter. The overall encounter and task times (Table 7) averaged slightly faster in almost every instance for the treatment group than that of the control group. Task times were faster in the control group for the medical history task, which can be explained by the fact that not all providers completed the task. As such, the overall mean was artificially reduced for the control group. Finally, SDs generally clustered more closely around the mean for tasks in the treatment group than that in the control group.

A benefit of patient care efficiency might be related to the fact that the providers had more time to conduct discussions on patient education. The mean for every educational task was higher for the treatment group than for the control group. This difference likely contributed to the higher percentage of the encounter time overall (ie, time spent on the 3 tasks by the treatment group).

The emphasis on task completion is also relevant for an analysis of patient education. Although efficient and effective patient care is not always correlated, we argue that time is another indicator of a more *effective* use of provider time in the study. The importance of patient education to providers underpins this analysis. If the efficient use of time leads to the discussion of additional important information (ie, patient education), then the encounter can be considered more effective.

### Satisfaction, Common Ground, and Performance

The quantitative analysis of the posttest questionnaire examined secondary user perceptions of satisfaction, common ground, and performance. For the posttest questionnaire, a Cronbach alpha reliability analysis (Table 8) was performed on all the subscales using SPSS 23. To improve the reliability of the satisfaction subscale, 2 items were removed. This removal increased the reliability to ≥.80, which is good reliability [35]. Reliability for the common ground and performance subscales were ≥.80 and .90, respectively, which are good and excellent reliabilities, respectively [35].

**Table 7:** Descriptive statistics for encounter and task times for the 6 participants in the control group and 6 participants in the treatment group.

Task		Control group			Treatment group			
		Mean (SD)	Median	Min-Max		Mean (SD)	Median	Min-Max
Encounter^a^		0:12:45 (0:06:19)	0:11:09	0:06:35-0:25:05		0:12:22 (0:02:38)	0:12:30	0:08:47-0:15:40
**Rash^b^**		0:02:52 (0:02:33)	0:02:08	0:01:12-0:08:02		0:02:40^c^ (0:01:36^c^)	0:02:02^c^	0:01:44^c^-0:05:53^c^
	Education^d^	0:00:18 (0:00:44)	0:00:00	0:00:00-0:01:50		0:00:19^c^ (0:00:31^c^)	0:00:00^c^	0:00:00^c^-0:01:13^c^
**Med reconciliation^e^**		0:03:03 (0:00:58)	0:02:37	0:02:33-0:05:02		0:02:24^f^ (0:01:05^f^)	0:02:11^f^	0:01:09^f^-0:04:14^f^
	Education^d^	0:00:17 (0:00:29)	0:00:00	0:00:00-0:01:13		0:00:36^f^ (0:00:58^f^)	0:00:00^f^	0:00:00^f^-0:02:14^f^
**History^g^**		0:01:25^f^ (0:01:43^f^)	0:00:57^f^	0:00:00^f^-0:04:10^f^		0:02:46^f^ (0:01:38^f^)	0:02:23^f^	0:00:54^f^-0:05:04^f^
	Education^d^	0:00:00^f^ (0:00:00^f^)	0:00:00^f^	0:00:00^f^-0:00:00^f^		0:00:23^f^ (0:00:57^f^)	0:00:00^f^	0:00:00^f^-0:02:21^f^
Total time spent on required tasks^h^		0:07:57 (0:02:39)	0:07:40	0:04:41-0:11:25		0:09:11 (0:01:49)	0:09:12	0:06:45-0:11:16

^a^The percentage of the total encounter time spent on the three tasks: control group=67%; treatment group=76%.

^b^Rash: time discussing the problem.

^c^Indicates a task for which partial common ground was achieved.

^d^The mean times for any patient education related to a specific task.

^e^Med reconciliation: time discussing medication reconciliation.

^f^Indicates a task for which common ground was achieved.

^g^History: time discussing medical history.

^h^The total time spent on the three tasks: Rash, med reconciliation, and history.

**Table 8:** Subscale reliability analysis.

Characteristics	Cronbach alpha	Item numbers
Satisfaction	.824	3
Common ground	.892	6
Performance	.981	4

**Table 9:** Descriptive statistics for the posttest questionnaire subscales.

Group		Satisfaction	Common ground	Performance
**Control**				
	Mean (SD)	5.83 (1.44)	5.94 (1.25)	5.83 (1.54)
	Minimum-maximum	3.00-7.00	3.83-7.00	3.00-7.00
**Treatment**				
	Mean (SD)	5.67 (1.01)	5.75 (0.43)	5.25 (0.98)
	Minimum-maximum	4.33-6.67	5.17-6.17	4.00-6.00

The descriptive statistics for each subscale (Table 9) show higher means for the control group than for the treatment group, indicating that members of the control group perceived that they had a higher level of satisfaction, common ground, and performance than members of the treatment group. SDs show better clustering around the mean for the treatment group on each subscale, which could indicate a better consensus among treatment group members, reflecting a more accurate evaluation of satisfaction, common ground, and performance in the treatment group than in the control group.

## Discussion

### Overview

The experimental study provided substantial insight on grounding in the context of a face-to-face clinical interaction. The research questions were comprehensively addressed during the study. Grounding was better in the treatment group, indicating that the idea of integrating collaboration mechanics into interactive technology designs with the intent to improve grounding has merit. The specific findings for each research question are listed below:

1. Research question 1 finding: The experimental study results indicated that smartphone apps designed using collaboration mechanics support grounding between primary and secondary users during face-to-face collaborations and can facilitate complete common ground. The success of grounding with them is task-dependent.
2. Research question 2 finding: The experimental study results indicated that smartphone apps designed to support grounding have the potential to positively impact secondary user satisfaction, performance, and perspective about the primary user’s commitment to the collaboration.

### Explanation of Outcomes

#### Similar Outcomes

Grounding occurred in both groups regarding the medical history task. However, the time analysis shows that the minimum time on task for the control group was 0 seconds, whereas the treatment group’s minimum time was 54 seconds. Furthermore, none of the providers in the control group conducted patient education related to the medical history task, which was contrasted with a mean time in the treatment group of 23 seconds. Thus, even though grounding for this task occurred in both groups, it occurred throughout the treatment group with the additional benefit of including patient education. This aspect of the time analysis indicates that the encounters in the treatment group were more effective than those in the control group because all treatment group members accomplished the required task and made time for education, whereas those in the control group did not.

#### Control Group Outcomes

In the control group, grounding did not occur for problem identification or medication reconciliation. Participants were not able to confidently identify any aspect of the rash other than it had occurred and had healed. Although several participants were able to exclude some environmental causes during their discussions with the trained patient actor (eg, no recent changes to medications), the grounding that did occur involved future patient action if the rash recurred (ie, contact the office immediately). One participant described the difficulty of evaluating the no longer visible rash as:

The rash, that was difficult because once he described the rash, I thought of probably three things it could have been. So, maybe that was probably about an eight [out of 10] difficulty just because it’s not there anymore. So, I can’t treat something or even tell him what it is without having seen it. I can’t treat something I can’t see.

Participant 3-C (APRN)

Less grounding occurred in this instance, because the rash was gone, and the trained patient actor’s verbal description was of limited utility.

Regarding medication reconciliation, there was no indication that the providers were able to glean enough information to be confident that they had correctly identified the patient’s medication regime. On one hand, medication reconciliation was the most challenging task for participants in the scenario. The lack of patient medication knowledge inhibited decision making about the patient’s health care. One participant commented:

He didn’t know his medication doses and timing, so I was worried that there was a high risk for error in assuming [that] what he was telling me was right. It was difficult to make recommendations or a plan without knowing what those medications were.

Participant 3-C (APRN)

On the other hand, the preliminary work for the study indicated that providers typically have a substantial amount of experience with patients who lack detailed knowledge about their own medications. This is consistent with what was observed during the study.

Because participants were accustomed to dealing with a lack of medication information, they knew what questions to ask to devise workarounds. Consequently, they were able to create a plan of action with the trained patient actor that would result in getting the correct information. However, even with workarounds, significant treatment delays could be expected as 1 participant acknowledged:

What I didn’t know was his medications, and I didn’t want to guess. I needed that information, but I just have to find it from another source or ask him next time to bring his medications.

Participant 2-C (APRN)

The participants indicated that this situation—patient lack of knowledge about their medication details—was typical of encounters with new patients.

It was apparent that grounding did not occur in the control group for the medication reconciliation task. Although there were successful joint actions between the participants and trained patient actor that culminated in planning activities to get the correct information, these did not enhance the quality of the encounter. In fact, the lack of common ground made the encounters distinctly inefficient in the control group.

#### Treatment Group Outcomes

In the treatment group, grounding occurred for the problem identification and medication reconciliation tasks. Although participants could not determine the cause of the rash or swab it for testing, the picture sparked deeper engagement with the trained patient actor about the ailment and allowed participants to exclude some diagnoses. It also seemed to improve general confidence among participants about their interactions with the trained patient actor. The availability of an image of the rash was helpful, as highlighted by this participant:

I like that he took pictures of his wound. It would have been, like I told him, helpful in the future to actually be seen when the situation is acute versus resolved but taking that photo when it was an active rash was helpful—at least would be helpful—to the physician.

Participant 6-T (RN)

Although grounding was not complete for this task, treatment group participants were typically more willing to share detailed information about this type of wound with the patient. This is noteworthy because rashes are often a difficult clinical issue, so any improvement to communication about such a problem is important. The ability to view an image of the rash clearly aided the communication.

**Figure 7:**
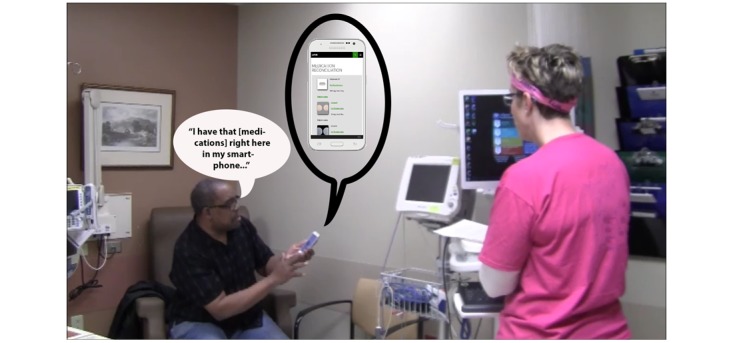
The patient provides accurate medication information by using the prototype to augment his memory, making the attainment of common ground possible.

Use of the prototype resulted in common ground being obtained during the medication reconciliation task. Treatment group participants were confident at the end of the interaction that critical and accurate information about the medications had been relayed to them. This allowed them to probe deeper with specific questions or spend more time on patient education. See Figure 7 for an example of the interaction between the trained patient actor and a study participant during a treatment group simulation.

Treatment group members trusted the information provided from the prototype. For example, participant 5-T (APRN) remarked that:

...my gut reaction is that it’s accurate and it’s a tool that can be shared between the patient and the provider.

Participant 5-T (APRN)

The information obtained during the medication reconciliation task was of good enough quality to support medical decision making. In another example, Participant 6-T (RN) felt that the hospital could dispense medications to a patient admitted for an overnight stay with the level of detail provided by the trained patient actor.

Successful joint actions between the trained patient actor and participants, leading to common ground, occurred during all tasks for the treatment group. However, the completeness of common ground and relevance of the prototype did vary by task. The prototype supported successful joint action and partial common ground for the problem identification task and complete common ground for the medication reconciliation task. The prototype was unnecessary for accomplishing common ground during the medical history task.

Overall in the treatment group, joint actions were comprehensive enough that participants could make plans for treatment based on the details obtained during the interview. For example, at the end of the encounter, the participants had enough accurate information about medications to maintain the current prescriptions (because they were the correct medicine at the proper dosage for the scenario). This was not the case in the control group. The lack of certainty about medications meant that participants could not evaluate if they were working for the ailments depicted in the scenario.

Although the posttest questionnaire implies less satisfaction among members of the treatment group, the richness and depth of the findings of the qualitative analysis indicate the opposite and provide a more comprehensive view of the data. The overall qualitative analysis clearly indicated that providers in the treatment group were more satisfied than their peers in the control group. The completeness of information and its contribution to a more successful encounter becomes obvious (because of the qualitative analysis) as reflected in the comments of 1 participant:

That information that you have to have to make decisions, we didn’t have to spend a lot of time figuring that out [because of the prototype]. We were able to quickly get all of that and then move onto here’s what we’re going to do about the problems that you have, the issues that you have, and go from there. So, we had more time for that, rather than just trying to figure out the historical data.

Participant 2-T (Doctor)

For the treatment group, the analysis of satisfaction expressed by the providers combined with the descriptive statistics for the performance of the tasks, indicate that treatment group encounters were more efficient and effective than those in the control group.

The qualitative analysis was a rich source of data and provided detailed insight for answering the research questions. The quantitative analysis of the task performance supported the conclusions of the qualitative analysis. According to these measures, the use of the mobile phone optimized common ground for members of the treatment group. In contrast, the results from the posttest questionnaire were a bit perplexing. For example, research about the impact of introducing examination room computers to the patient-provider encounter showed a higher degree of satisfaction among patients after the introduction of computers [36]. One would expect a similar outcome for clinicians in this research. It is unclear why the *perspectives* of the participants in each group, as assessed using the posttest questionnaire, were reversed.

Although the posttest questionnaire implied that the control group’s perceptions were more favorable than that of the treatment group, this analysis was clearly not supported based on the strong positive responses of the treatment group members during the simulations and semistructured interviews. Poor clustering around the mean for satisfaction, common ground, and performance for the control group on the posttest questionnaire indicated less consensus among them about the variables than for the treatment group.

There are other factors, such as the distinction between the UX and usability, that may help explain how and why the differences in perception occurred. The UX is a person’s emotional response to an interactive system, whereas usability is a technical aspect that emphasizes how well a person can use a system. The placement of a patient-managed technology between the provider and the trained patient actor may have subtly and negatively transformed provider perceptions in the treatment group. Although providers in the treatment group liked the usability of the system and found it helpful, they might have perceived it as creating a barrier between themselves and the trained patient actor in some way.

This idea of a barrier makes sense when considering the emotional nature of the UX and the technical nature of usability. Moreover, the control group was largely made up of primary care professionals who look at patients holistically and longitudinally. The treatment group was largely made up of specialists who may emphasize a procedural perspective because they look at patients for specific ailments and patient relationships tend to be much shorter than the relationships cultivated by their primary care peers. However, this distinction is a minor point because the overall analysis of the data clearly indicates that the performance was better and the UX was more satisfactory for the treatment group.

#### Unanticipated Outcomes

Finally, a few unanticipated outcomes of insight were gleaned. The first was the possible impact of the technology on the perspectives of the provider. For example, the semistructured interviews revealed that the providers in the treatment group were happy with the outcome of the encounter and felt that the information exchange was better than usual. However, they did not record a level of satisfaction higher than their peers in the control group on the posttest questionnaire. The disparity indicates the need for a closer look at the potential impact of patient-centered technology.

Another insight was the willingness of providers to become responsible for clinical data from nonclinical sources. An early assumption during the requirement-gathering process for designing the prototype was that providers might not be willing to help manage these type of data. Nothing in the following stages of preliminary work or the experimental study suggested any reluctance on the part of providers to interact with these type of data. Rather, the providers considered the data trustworthy or, at least, as trustworthy as other types of patient-provided information that they hold in high regard.

### Implications of Results

The medication reconciliation task produced the strongest example of grounding in the research, which should not be surprising, as study participants indicated that 50% to 90% of their patients do not have accurate medication information with them during a real-world encounter. This lack of accurate information suggests a gap in knowledge and in patient-centered tools to aid patient recall. As the experimental study demonstrated, a tool that provides relevant medication information to providers can facilitate common ground. The prototype’s interface provided external representations of medications (ie, images of each medication) that were clear objects of interest to secondary users. The representations allowed participants in the treatment group to quickly collaborate with the trained patient actor about his medications, regardless of the secondary user’s interaction style with the prototype.

### Limitations

The scope of the research was restricted to the relation between usability and common ground within an mHealth setting. The research initially relied on a thematic analysis according to common ground theory. The research culminated in a small sample experimental study using a simulation. Consequently, there are limitations for generalizability.

Form factor and device functionality were relevant to the experimental study regarding the simulated patient-provider interactions. In addition, the sample size was too small for hypothesis testing. Therefore, the results may not be generalizable to collaborations outside the scope of short-duration, face-to-face clinical encounters. Nonetheless, even with a small sample size, the groundbreaking nature of the study offers value to the health care community because it emphasizes the patient as a knowledgeable collaborator—and as one who (through mHealth) can share their personal health information in a manner that improves the overall efficiency and effectiveness of provider care.

### Future Research and Recommendations

This study is the first that we know of to explore common ground using both primary and secondary UXs. The results indicate that there is a need to consider primary and secondary users when designing a single system for information sharing between those with expert and nonexpert levels of knowledge. To improve generalizability, the study should be replicated with a larger sample and providers who work in the same hospital or practice. It would be best to limit the study participants to 1 skill or specialty.

Limiting participants has several advantages for ecological validity. For example, EMRs and intake sheets with simulated patient data can be created using the systems common to the organization. This will increase realism in the simulation for participants. Furthermore, with the standardized procedures, training, and tools common to a single organization and specialty, confounding variables can be limited.

Finally, the prototype used for the simulation should be a mobile phone app running on the device rather than a responsive design website. There was some latency accessing the website based on location and network quality, which caused small delays. A native app running on a mobile phone mitigates the impact of a slow running website and will more accurately capture shorter encounter times because of the faster load times for a native app.

### Summary

During the preliminary work, the priorities that emerged for providers in the type of encounter simulated were to conduct medication reconciliation, problem identification, and then medical history. The priorities, regarding the technology and its efficacy as a tool to facilitate communication, were supported during the experimental study. For example, peak usefulness of the prototype was demonstrated whenever participants attempted to glean highly clinical data (ie, detailed medication information) from the primary user, who is typically a nonclinical source for such information.

Furthermore, participants in the treatment group had improved confidence because the rash image was available. Finally, although technology did not improve or detract from grounding for members of the treatment group during the medical history task when compared with the control group, it seemed to create efficiencies for the overall encounter that allowed all members of the treatment group to complete the task (whereas all members of the control group did not).

The alignment of the perceptions of the relative importance of the respective tasks from providers to the actual creation of common ground during the experimental study should be interpreted as providing a level of awareness for design decisions regarding the allocation of time and other resources. If tasks are difficult to accomplish (ie, medication reconciliation), then attempts to promote grounding using technology may be a good use of resources, while it may be a poor use of resources for easy tasks (ie, medical history). This identification of importance and the relation to common ground is a vital insight for the overall body of secondary user research.

Regarding outcomes in the different experimental groups, it is not that grounding did not occur in the control group—it did. Rather, it is the relevance of the common ground achieved for solving the problem at the center of the need for communication that is at issue. Participants noted that the attainment of common ground during a first encounter with a new patient was frequently rare in real life. They also indicated that they would support patients’ uses of smartphones as a tool by which to improve face-to-face communication during encounters. This participant highlights why smartphone use would be acceptable:

The easiest thing about sharing information with this patient was his ability to use technology to show me, so that we were both on the same page. As opposed to [a verbal] description, [where] I paint my own [picture]. Then, we’re both on apples to apples, instead of me trying to paint in my head what he’s describing.

Participant 4-T (RN)

This study indicated that well-designed systems that deepen the engagement of patients in their own health care while improving near-term communication with providers have a place in HIT.

The provider responses in the treatment group reinforced design decisions about the information that should be made available to secondary users. Overall, the utility of patient-controlled devices during a first encounter with a patient depends as much on the difficulty for the provider of normally obtaining the information intended to be shared, and its impact upon immediate decision making, as on the HCI design decisions.

Medication reconciliation is a difficult task that is necessary for successful treatment decisions. In real life, inconclusive reconciliation is so routine that it is expected among providers. The introduction of technology to mitigate a patient’s personal lack of knowledge has the potential to create useful common ground within the dyad for this type of complex health care task. This observation is another critical insight for the overall body of secondary user research.

### Conclusions

The experimental study is one of the first studies to directly demonstrate that not all secondary UXs are meaningful for design. For example, grounding occurred during the medical history task in the control and treatment groups. Any marginal improvement due to implementation of technology, where grounding occurs regardless of technology, is probably not worth the effort. The fact that common ground was only completed in the treatment group, and during medication reconciliation (an essential enabler for health care decision making), indicates that an investment in the secondary UX for high payoff tasks is valuable.

Combining the notions of common ground, HCI design, and smartphone technology resulted in a prototype that improved the efficiency and effectiveness of face-to-face collaboration for secondary users with the primary user. The prototype clearly facilitated a higher quality of information exchange than normal. Thus, the investigation substantiated the notion that properly designed interactive systems have the potential to facilitate common ground while providing a satisfactory secondary UX.

## Appendix

Multimedia Appendix 1 Experimental study scenario.

Multimedia Appendix 2 Checklist to assess treatment fidelity.

Multimedia Appendix 3 Posttest questionnaire.

Multimedia Appendix 4 Posttest semistructured interview guide.

## Abbreviations

APRN: advanced practice registered nurse
EMR: electronic medical record
HCI: human-computer interaction
HIT: health information technology
mHealth: mobile health
PHR: personal health record
RN: registered nurse
UX: user experience
