# Supplementary material for: Simulated Clinical Encounters Using Patient-Operated mHealth: Experimental Study to Investigate Patient-Provider Communication
Source: JMIR Mhealth Uhealth. 2018 Nov 1;6(11):e11131. doi: 10.2196/11131 (PMC6238098; doi:10.2196/11131)
Supplement: Multimedia Appendix 1 [file mhealth_v6i11e11131_app1.pdf]

Multimedia Appendix 1: Experimental Study Scenario

This is meant to represent a routine scheduled visit as in your normal practice. You have another patient scheduled after you finish with the one you are about to see.

You are seeing a patient for the first time who switched to you from another provider. The patient has Type 2 diabetes. The patient also suffers from obesity, hypertension, and high cholesterol. The patient reports recently having a rash. The rash healed before the appointment, so it is no longer visible.

You are seeing a 56-year-old male named Dennis.

The patient’s height is 5’11” and weight is 263 lbs.

The patient’s diabetes (Hemoglobin A1C: 6.6) appears to be under control with medication. The patient’s cholesterol is available (LDL: 150, HDL: 60). Today, the patient’s temperature (98.6), heart rate (83), respiration rate (16), and blood pressure (120/80) have been taken by the Medical Assistant. However, medication reconciliation has not been performed.

You have three tasks to perform with this new patient (do them in the order you think appropriate):

- Go over the patient’s problem,
- Update what you know about the patient’s medical history, and
- Conduct medication reconciliation.
