# Supplementary material for: Simulated Clinical Encounters Using Patient-Operated mHealth: Experimental Study to Investigate Patient-Provider Communication
Source: JMIR Mhealth Uhealth. 2018 Nov 1;6(11):e11131. doi: 10.2196/11131 (PMC6238098; doi:10.2196/11131)
Supplement: Multimedia Appendix 2 [file mhealth_v6i11e11131_app2.pdf]

# Multimedia Appendix 2: Checklist for Treatment Fidelity

## Without the Smartphone

When describing the rash, make the following statements:

- *When discussing the location of the rash*: "It was a big rash on my right elbow and arm."
- *When describing the rash*: "They were like blisters." And "Some would break and some were filed with fluid stuff. It was clear, I think."
- *When discussing how long the rash lasted*: "It lasted about three weeks and then went away by itself. It's been gone a week or so."
- *When asked about the cause of the rash*: "I don't know. I use a lot of chemicals, cleaning solvents and stuff, at work. It could be anything, I guess."

When describing past medical history, make the following statements:

- *When asked about blood pressure history*: "I started my job about six years ago and I started having blood pressure problems the next year, so about five years."
- *When asked about diabetes*: "I started taking diabetes medication about two years ago."
- *When asked about cholesterol*: "The cholesterol thing started about 2–3 years ago."

When describing medications, make the following statements:

- *When discussing the total number of medications*: "I know I take four medicines. Two for my diabetes, one for my cholesterol, and one for my blood pressure."
- *When discussing Lisinopril*: "I take Lisinopril, once a day, 10 milligrams for my blood pressure."

- *When discussing Metformin ER*: “One of the ones for my diabetes is Met, Met, Met something. It’s oval shaped and I take it twice a day. It’s 500 milligrams. I remember that because that’s the biggest number.”
- *If they follow-up by asking if its Metformin*: “That sounds right.”
- *If they follow-up by asking if it’s something else*: “That doesn’t sound familiar.”
- *When discussing Atorvastatin*: “It’s Ater-Vas-A-Tin or something like that. I take it at night. I think that’s my cholesterol pill.
- *When discussing Glipizide*: “The other one for my diabetes begins with a G. It’s a white round pill. It’s a real small dose and I take it with meals. That’s about all I remember.”
- *If they follow-up by asking if its Glipizide*: “That sounds right.”
- *If they follow-up by asking if it’s something else*: “That doesn’t sound familiar.”
- *When asked where you get your medications*: “CVS.”

## With the Smartphone

When describing the rash, make the following statements:

- *When describing the rash*: “It was on my right arm and elbow. I took a picture of it. Would you like to see it?”
- *When discussing how long the rash lasted*: “It lasted about three weeks and then went away by itself. It’s been gone a week or so.”
- *When asked about the cause of the rash*: “I don’t know. I use a lot of chemicals, cleaning solvents and stuff, at work. It could be anything, I guess.”

When describing past medical history, do the following:

- Refer to the information in the smartphone, but only give it to the provider if they ask to it.
- *When asked about blood pressure history*: “It’s in my phone. Let me check.”
- *When asked about diabetes*: “It’s in my phone.”
- *When asked about cholesterol*: “Let me check my phone.”

When describing medications, make the following statements:

- *When discussing the total number of medications*: “I know I take four medicines. Two for my diabetes, one for my cholesterol, and one for my blood pressure.”
- *When asked for more detail about medications*: “I don’t remember all of their names, but I have a list in my phone. Would you like to see it?” (If they do not want to see the smartphone, then refer to the information in the smartphone as you tell them the medications.)
- *When asked where you get your medications*: “CVS.”
