# Supplementary material for: Simulated Clinical Encounters Using Patient-Operated mHealth: Experimental Study to Investigate Patient-Provider Communication
Source: JMIR Mhealth Uhealth. 2018 Nov 1;6(11):e11131. doi: 10.2196/11131 (PMC6238098; doi:10.2196/11131)
Supplement: Multimedia Appendix 3 [file mhealth_v6i11e11131_app3.pdf]

Multimedia Appendix 3: Posttest Questionnaire

Demographics

1. Did the patient use a smartphone during the interaction?
   a. Yes
   b. No
2. What is your job?
   a. Physician
   b. RN
   c. Nurse Practitioner
   d. Physician Assistant
   e. LPN
   f. Other (please specify)

3. What is your age?
   a. 18–24
   b. 25–34
   c. 35–44
   d. 45–54
   e. 55–64
   f. 65–74
   g. 75 or older
4. What is your gender?
   a. Female
   b. Male

5. How long have you been a practicing clinician?
    a. < 1
    b. 1–5 years
    c. 6–10 years
    d. 11–15 years
    e. 16–20 years
    f. 21–25 years
    g. More than 25 years
6. What type of practice are you in?
7. How often do you see patients?
    a. 1–3 days per week
    b. More than 3 days per week
    c. A few days per month
    d. I do not treat patients.
    e. Other (please specify)
8. What do you review before you see a patient? (Check all that apply.)
    a. The patient’s intake sheet that they filled out before the visit or when they arrived at the office.
    b. The patient’s electronic health/medical record.
    c. The patient’s paper medical record.
    d. Other (please specify)
9. What is your specialty?
10. Do you have a mobile phone?

a. Yes, I have a smartphone.
b. Yes, I have a “flip phone.”
c. No

11. Do you have a tablet computer?
    a. Yes
    b. No
12. How many hours a day do you spend on the Internet?
    a. <1 hour
    b. 1–2 hours
    c. 3–5 hours
    d. 6–8 hours
    e. 9+ hours
13. How many years have you been using computers?
    a. < 1 year
    b. 1–5 years
    c. 6–10 years
    d. 11–20 years
    e. 20 + years

Satisfaction (7-Point Scale)

14. I enjoyed communicating and sharing ideas using the available tools with my patient.
15. I enjoyed planning with my patient.
16. I would enjoy working with my patient and building a health plan using the available tool(s).

17. I am satisfied with the plan we just completed.

18. I wish we could change the plan we just completed.

Common Ground (7-Point Scale)

19. I found it difficult to keep track of the conversation.

20. During the conversation, I was able to focus on the task at hand.

21. My patient and I communicated well with each other.

22. Over time, I got to know my patient better.

23. Over time, my patient and I came to share more and more ideas about the project.

24. Over time, my patient and I shared more ideas about the available tools.

Performance (7-Point Scale)

25. Our teamwork was effective.

26. Our teamwork was time-efficient.

27. My patient and I produced a good amount of work together.

28. My patient and I produced a good quality of work together.
