# Supplementary material for: Simulated Clinical Encounters Using Patient-Operated mHealth: Experimental Study to Investigate Patient-Provider Communication
Source: JMIR Mhealth Uhealth. 2018 Nov 1;6(11):e11131. doi: 10.2196/11131 (PMC6238098; doi:10.2196/11131)
Supplement: Multimedia Appendix 4 [file mhealth_v6i11e11131_app4.pdf]

Multimedia Appendix 4: Posttest Semistructured Interview Guide

1. How do you think the interaction with the patient went? (Be prepared to probe more fully. If their comment focuses on the negative, ask for positive points and vice versa.)
2. How confident were you in the quality of information provided by the patient?
3. What are your other thoughts about the quality of information shared during this interaction?
4. What was the easiest thing about …? (The thing selected will be based upon the prior answers. Ask them to rate it on a scale of 1–10 with 1 being very easy)
5. What was the hardest thing about ...? (The thing selected will be based upon the prior answers. Ask them to rate it on a scale of 1–10 with 1 being very easy)
6. Do you think a patient having their health information on a smartphone is useful in an interaction like this?
7. What do you think the impact of a patient referring to their health information on a smartphone during an interaction with you would be on time management?
8. Would you recommend to a patient that they store relevant health information on a smartphone and refer to it, or let you review it, during an appointment with you? Why/why not?
9. Overall, how satisfied were you with the interaction?
